# Design and Synthesis of Peptide‐Polyester Conjugates for Cell‐Mediated Scaffold Degradation

**DOI:** 10.1002/adhm.202504885

**Published:** 2026-02-21

**Authors:** Korina Vida G. Sinad, Natasha K. Hunt, Srujan Singh, Kelly B. Seims, Yingjie Wu, E. Thomas Pashuck, Warren L. Grayson, Lesley W. Chow

**Affiliations:** ^1^ Department of Chemistry Lehigh University Bethlehem Pennsylvania USA; ^2^ Department of Bioengineering Lehigh University Bethlehem Pennsylvania USA; ^3^ Department of Chemical and Biomolecular Engineering Johns Hopkins University Baltimore Maryland USA; ^4^ Translational Therapeutics & Regenerative Engineering Center Johns Hopkins University School of Medicine Baltimore Maryland USA; ^5^ Department of Materials Science & Engineering Lehigh University Bethlehem Pennsylvania USA; ^6^ Department of Biomedical Engineering Johns Hopkins University Baltimore Maryland USA; ^7^ Institute for Nanobiotechnology Johns Hopkins University Baltimore Maryland USA; ^8^ Department of Materials Science and Engineering Johns Hopkins University Baltimore Maryland USA; ^9^ College of Health Lehigh University Bethlehem Pennsylvania USA

**Keywords:** biodegradable polymers, biomaterials, cell‐mediated degradation, peptides, proteases, tissue engineering

## Abstract

Biodegradable polyesters are promising biomaterials for tissue engineering. Polycaprolactone (PCL) is particularly attractive for orthopedic applications like craniofacial bone repair but does not degrade at the same rate as new tissue formation, which may compromise functional regeneration. To address this, we incorporated a protease‐cleavable peptide directly into the PCL backbone. A functional mass spectrometry approach was used to identify a fast‐degrading peptide (Fast) selectively cleaved by multiple cell types. Conjugates containing Fast or its scrambled control (ScrFast) were solvent‐cast with an RGDS‐PCL conjugate into disks. Including Fast and ScrFast peptides did not impair cell adhesion. Cy3‐labeling enabled real‐time quantification of degradation in the presence of collagenase or human mesenchymal stromal cells (hMSCs). After 21 days in collagenase, Fast‐PCL released 20.38 ± 2.17 nmol Cy3 (25.77% ± 3.70%) vs. 8.70 ± 0.92 nmol (11.31% ± 1.01%) for ScrFast‐PCL with respective mass losses of 22.1% ± 1.2%, and 18.9 ± 3.8%, indicating enzyme‐mediated degradation. Under hMSC‐mediated degradation, Fast‐PCL released 30.31 ± 3.18 nmol Cy3 (26.68% ± 2.17%) compared to 23.91 ± 2.13 nmol (18.97% ± 1.24%) from ScrFast‐PCL, indicating sequence‐dependent, cell‐directed resorption. This platform integrating protease‐sensitive peptides into the polymer backbone can be leveraged to couple scaffold remodeling to enhance tissue regeneration.

## Graphical abstract

This work describes polycaprolactone (PCL)‐based biomaterials engineered to degrade in response to cell‐secreted proteases. A fast‐degrading peptide (Fast) sequence is integrated into a PCL conjugate backbone to produce a biomaterial that is selectively degraded by multiple cell types compared to its scrambled control (ScrFast). This versatile design enables cell‐mediated biomaterial degradation that is coupled to specific cellular processes.

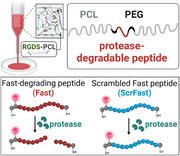

## 1 Introduction

Biomaterials for tissue engineering (TE) are designed to replace or restore the function of damaged or degenerated tissues by providing temporary physical support for cellular infiltration and tissue regeneration, while gradually being resorbed as new tissue forms [1, 2, 3, 4, 5]. Biodegradable thermoplastic polyesters like polycaprolactone (PCL), poly(lactic acid) (PLA), poly(glycolic acid) (PGA), and poly(lactide‐*co*‐glycolic acid) (PLGA) polymers are attractive scaffold materials due to their ability to degrade under physiological conditions [6, 7]. Moreover, they offer key benefits, such as batch‐to‐batch reproducibility, controllable chemistries and microstructures, and clinical use in FDA‐approved products [4, 8, 9, 10]. They also possess mechanical properties suitable for orthopedic applications like craniofacial bone and can be easily processed into patient‐specific geometries for complex defect repair [11, 12, 13, 14, 15]. For example, PCL 3D‐printed with decellularized bone into anatomically shaped scaffolds and implanted with human adipose‐derived stem cells significantly enhanced craniofacial bone healing in critical‐sized defects [16, 17]. While PCL‐based scaffolds provide adequate mechanical support, they demonstrate minimal degradation in vivo (∼24–48 months) that contrasts sharply with the typical timeline of bone healing (∼6 months) [6, 16, 18, 19, 20, 21]. Scaffolds that degrade too slowly may hinder tissue growth or leave voids once they degrade in the injury site post‐healing, compromising long‐term success [5, 16, 21, 22]. Conversely, those that degrade too rapidly risk losing mechanical integrity before sufficient tissue has formed [23, 24].

The degradation kinetics of biodegradable polyesters can be systematically controlled through chemical structure, composition, architecture, and processing [18, 24, 25, 26]. For example, blending low and high molecular weight PCL reduces its effective weight‑average molecular weight, shortening the degradation time without sacrificing mechanical integrity [27]. Copolymerization with faster‐degrading polyesters, such as PLA or PGA, is another approach to tune degradation rates by altering ester bond density and hydrophilicity [28, 29]. Scaffold processing methods, including adjusting porosity, pore size, and fabrication technique (e.g., solvent‐casting vs. 3D printing), also influence degradation by modulating water penetration and surface area exposed to hydrolysis [30, 31]. These strategies allow for pre‐set, bulk‐controlled degradation rates that produce scaffolds with predictable lifetimes under physiological conditions. This limits adaptability to patient‐specific healing dynamics, which are influenced by local factors (e.g., blood supply and infection), systemic factors (e.g., age, nutrition, and menopause), or mechanical factors (e.g., stress and movement) [32, 33, 34, 35, 36, 37, 38, 39, 40]. These complexities motivate the need for ‘smart’ scaffolds that respond to cellular activity to match scaffold degradation to tissue remodeling and regeneration processes.

Biological events, such as protease activity, have been leveraged to induce cell‐mediated biomaterial degradation [41, 42, 43, 44]. For example, cells secrete proteases like matrix metalloproteinases (MMPs) and the serine protease plasmin, to remodel their surrounding extracellular matrix (ECM) during migration and proliferation [45, 46, 47]. Synthetic hydrogel systems, particularly those composed of poly (ethylene glycol) (PEG), have been crosslinked with protease‐sensitive peptides for tunable degradation in response to cell‐secreted enzymes [48, 49, 50, 51]. Degradation rate can be controlled by changing the number of proteolytic cleavage sites and incorporating peptide sequences with varying protease specificity [45, 52]. Hydrogels have been the dominant material in this space due to their water‐swollen architecture that increases access to the protease‐sensitive peptides. However, their application is typically limited to drug delivery, injectable therapeutics, and soft tissue regeneration because of their relatively fast degradation rate (e.g., days to weeks) and low mechanical strength [53, 54, 55, 56, 57, 58].

Protease‐sensitive degradation has recently been expanded to more mechanically robust materials, such as solid thermoplastic polymers including polyurethanes and polyesters [59, 60, 61]. Fung and colleagues synthesized a linear poly(ester‐arylate) containing peptides with protease‐specific sensitivity [59]. Incorporating peptides within the polymer backbone resulted in protease‐specific polymer surface resorption, demonstrating the potential for employing protease‐guided degradation in hydrophobic thermoplastic polymers [59]. Similarly, elastase‐sensitive and collagenase‐sensitive sequences have been incorporated into polyurethane and poly(ether ester) systems, often aided by PEG to enhance hydrophilicity and protease accessibility [62, 63, 64, 65]. These studies underscore the potential for integrating protease‐responsive motifs into biodegradable thermoplastics to create solid polymeric biomaterials that degrade in concert with cellular activity.

**FIGURE 1:**
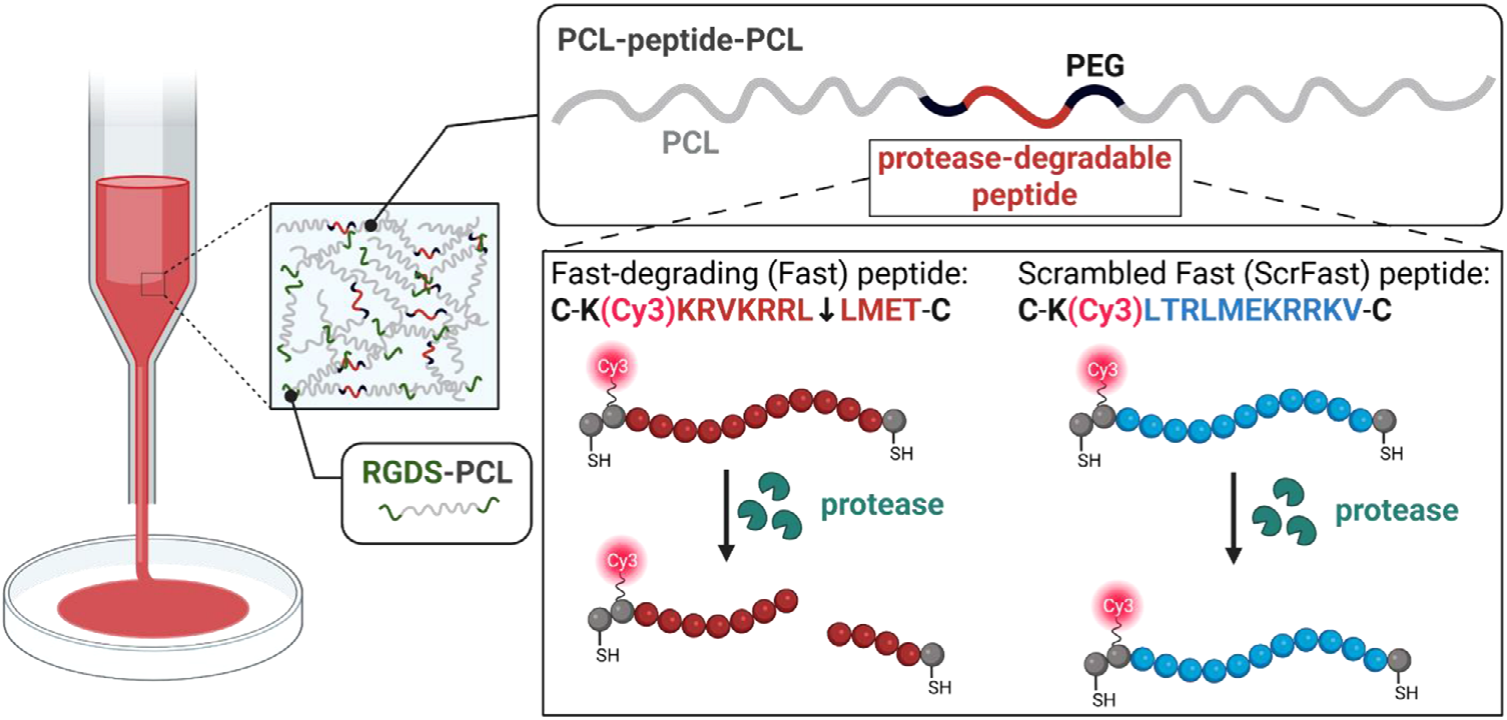
Schematic overview of biomaterial design. A fast‐degrading peptide (Fast) and the scrambled control (ScrFast) were integrated into the backbone of a maleimide‐functionalized polymer via Michael addition to synthesize Fast‐PCL and ScrFast‐PCL conjugates. Solid disks were prepared by solvent‐casting the conjugates. An RGDS‐PCL conjugate was included for cell adhesion. Protease‐mediated sample degradation was quantified using fluorescence measurements of solutions collected from samples incubated in protease‐containing media or in the presence of cells. Created with BioRender.com.

Here, we developed a strategy to create solid polymeric biomaterials that undergo selective, cell‐driven remodeling. Unlike prior systems that relied on known peptide substrates, we used a functional mass spectrometry‐based workflow to identify a peptide sequence that is cleaved by multiple cell types [66, 67]. We designed a robust synthetic approach that integrates the peptide within the backbone of PCL to create linear peptide‐PCL conjugates with PEG spacers flanking the peptide to facilitate protease access (Figure 1). Polymeric biomaterials fabricated with these conjugates exhibited sequence‐specific enzymatic cleavage in collagenase and in the presence of cells. This work introduces a versatile platform that combines proteomics‐driven peptide discovery with polymer conjugate design to create mechanically robust materials in which resorption is dictated by cellular activity.

## 2 Results and Discussion

### 2.1 Design and Synthesis of Protease‐Sensitive Peptide‐Polymer Conjugate

**FIGURE 2:**
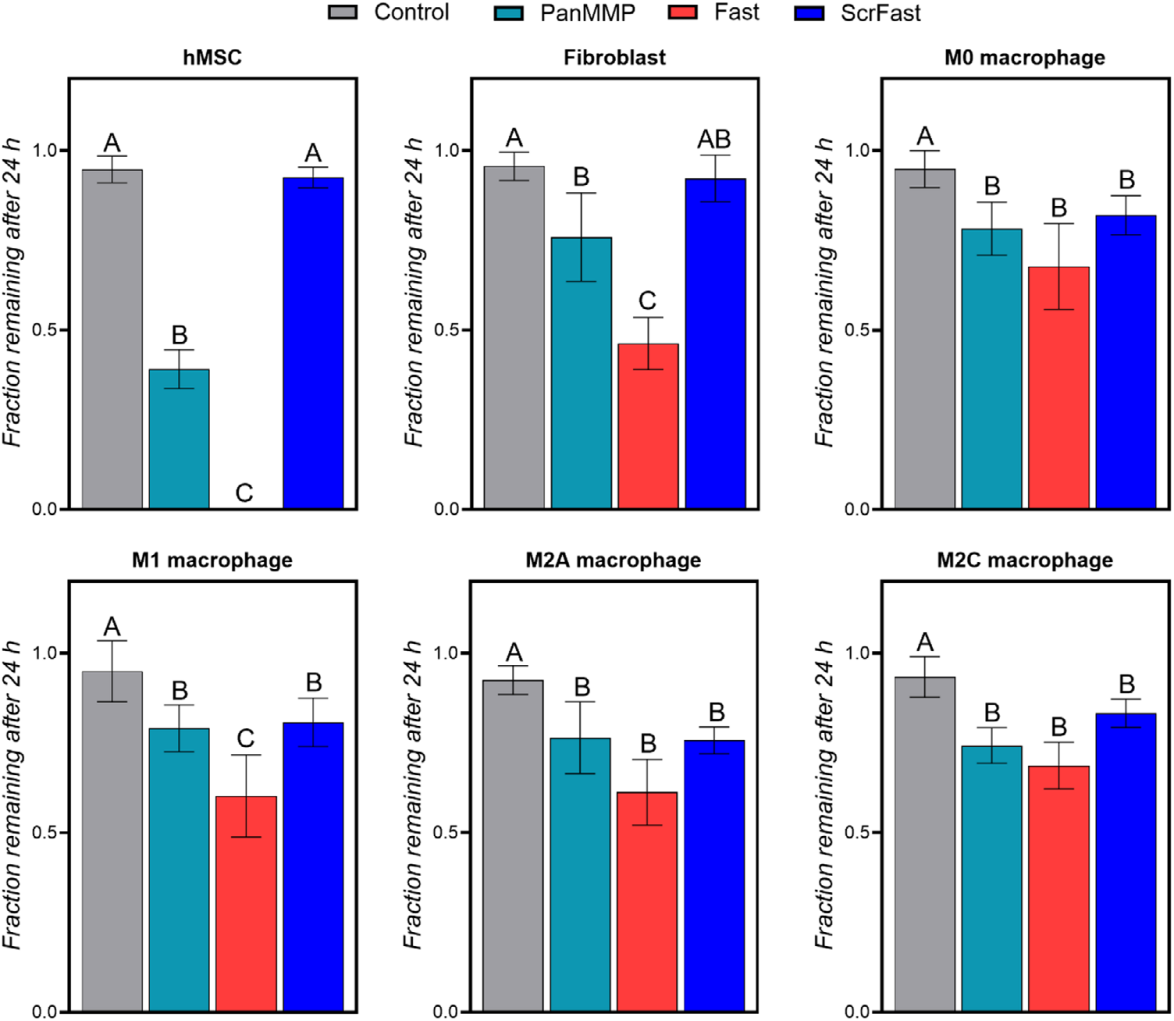
Fractions of candidate peptides remaining after 24 h of exposure to fresh media with hMSCs, fibroblasts, or different macrophage phenotypes. KRVKRRLLMET (Fast) demonstrated significant degradation compared to PPDKTSPEPA (Control) and KGPQGIWGQK (PanMMP) in the presence of hMSCs, fibroblasts, and M1 macrophages. The same behavior is observed when comparing Fast to the scrambled version (ScrFast). Data presented as mean ± SD (*n* = 3–8 samples per group). P‐values were calculated using one‐way ANOVA followed by Tukey's or Dunnett's T3 post‐hoc tests, as appropriate. Statistical analyses were performed separately for each cell type. Different letters within a cell type indicate statistically significant differences (*p* < 0.05).

We applied a functional mass spectrometry approach to identify novel protease‐cleavable peptides with broad relevance across multiple cell types [66, 67]. This method captures the cumulative enzymatic activity of both soluble and membrane‐bound proteases secreted by human cell types, allowing for the discovery of peptide substrates that are more biologically representative than canonical MMP‐degradable sequences like GPQGIWGQ (PanMMP) [66, 68]. Within this peptide library, we identified a peptide sequence, KRVKRRLLMET (Fast), that exhibited rapid proteolytic cleavage after 24 h by human mesenchymal stromal cells (hMSCs), fibroblasts, and M1 macrophages compared to KGPQGIWGQK containing the PanMMP sequence and a non‐degradable control sequence PPDKTSPEPA (Control) (Figure 2). We also synthesized a scrambled version of the Fast peptide, LTRLMEKRRKV (ScrFast), in which the positions of uncharged amino acids (L, M, V) were swapped with charged amino acids (E, K, R) to ensure the physiochemical properties of the scrambled peptide were significantly different. In contrast to Fast, ScrFast exhibited a significantly higher fraction remaining when exposed to hMSCs, fibroblasts, and M1 macrophages for 24 h (Figure 2). These results confirmed that degradation was driven by precise protease recognition rather than general peptide susceptibility. The Fast peptide therefore provided a model substrate to demonstrate the feasibility of engineering solid polymeric biomaterials capable of cell‐driven, sequence‐specific remodeling. ScrFast was selected as the negative control in subsequent studies.

We integrated the Fast and ScrFast peptides into a PCL conjugate backbone to compare sequence‐specific protease‐mediated sample degradation. Fast and ScrFast peptides were modified with a cysteine group on both termini to allow facile conjugation to polymers following thiol‐Michael addition. A water‐soluble Cy3 fluorophore was added to the peptide to monitor degradation via release of fluorescence into surrounding media. Successful synthesis and purification of the modified Fast and ScrFast peptides were confirmed via high performance liquid chromatography (HPLC), matrix‐assisted laser desorption/ionization time‐of‐flight mass spectrometry (MALDI‐ToF MS), and proton nuclear magnetic resonance spectroscopy (^1^H NMR) (Figures S1 and S2).

Cy3‐modified Fast and ScrFast peptides were coupled to maleimide‐functionalized PCL to create linear peptide‐PCL conjugates, Fast‐PCL and ScrFast‐PCL, respectively (Figure S3). Incorporating peptides into the backbone of hydrophobic polymers like PCL can reduce proteolytic access. Proteolytic cleavage of peptides requires access to the active site of enzymes [69, 70]. We therefore flanked the peptides with flexible, hydrophilic PEG spacers to increase steric accessibility of the protease‐substrate peptides [62, 63, 64]. ^1^H NMR confirms the presence of all expected components in the modified PCL systems, including characteristic resonances from the PCL backbone, PEG segments, and peptide moieties, supporting successful incorporation at the compositional level (Figures S3 and 3). However, NMR provides ensemble‐averaged chemical information and cannot unambiguously resolve chain architecture or distinguish fully conjugated multiblock species from partially modified polymers. We therefore used diffusion‐ordered NMR spectroscopy analysis to show that PCL (δ = 4. 07, 2.35, 1.63, and 1.40 ppm) and PEG (δ = 3.59 ppm) signals co‐diffuse at similar diffusion coefficients for Fast‐PCL and ScrFast‐PCL conjugates, confirming the formation of block copolymers rather than a physical mixture of homopolymers (Figure S5). The pronounced changes in solubility following conjugation also provided qualitative evidence of chemical modification of the PCL backbone. While PCL readily dissolves in dichloromethane (DCM), the peptide‐PCL conjugates are no longer soluble in this solvent and instead require more polar solvents such as DMF despite the PCL segments having limited solubility in DMF.

**FIGURE 3:**
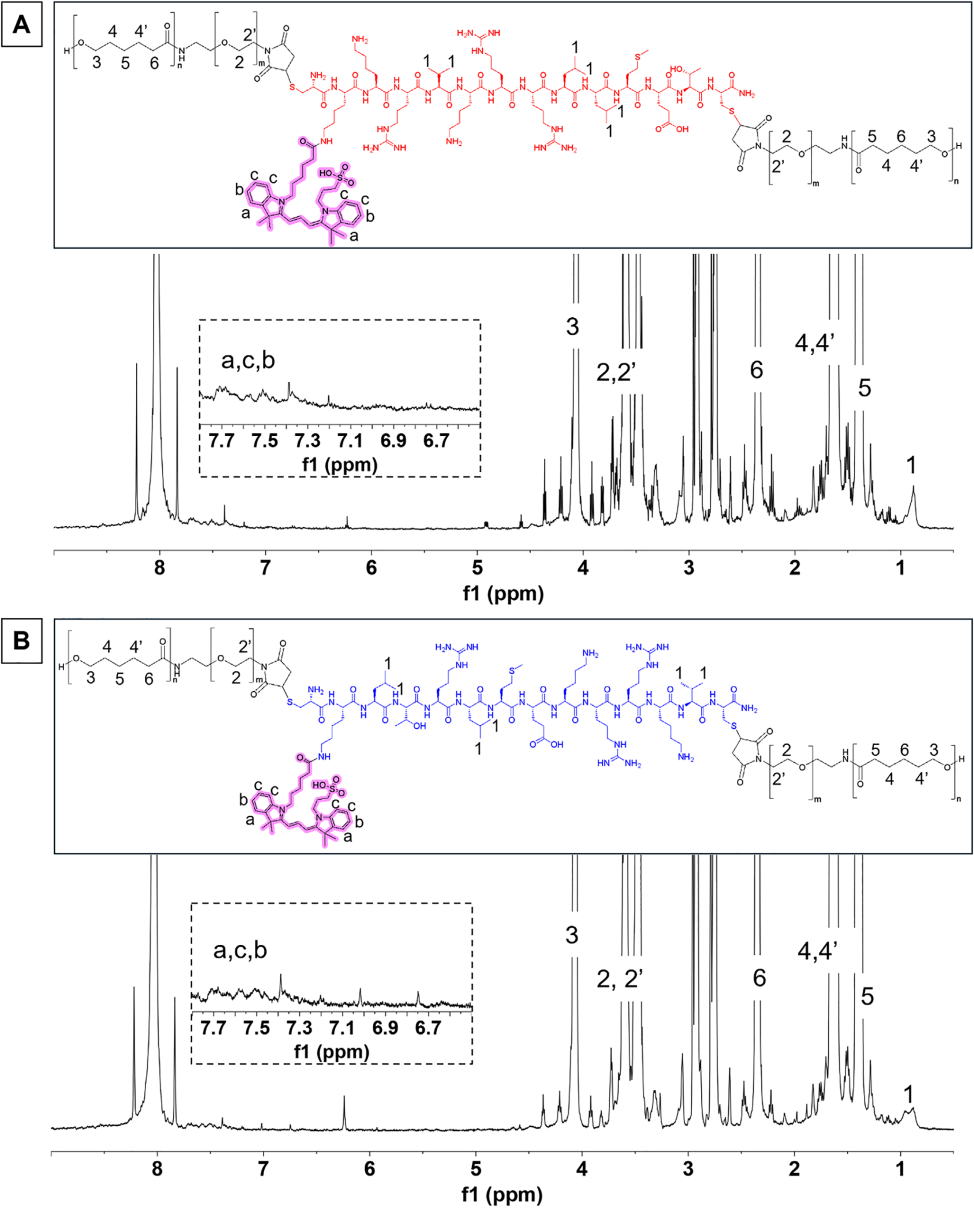
Representative ^1^H NMR spectra (500 MHz, DMF‐d_7_) and corresponding chemical structures of synthesized peptide‐PCL conjugates: (A) Fast‐PCL and (B) ScrFast‐PCL: δ = 7.71 (m, 2H, Ar─H, (a), 7.54 (m, 4H, Ar─H, c), 7.37 (m, 2H, Ar─H, (b), 4.07 (t, 912H, ─O─CH_2_─, 3), 2.35 (t, 912H, ─CO─CH_2_─, 6), 1.63 (m, 1824H, ─CH_2_─, 4/4’), 1.39 (m, 912H, ─CH_2_), 3.59 (s, 872H, ─O─CH_2_─CH_2_─, 2/2’), and 0.89 (m, 18H, ─CH_3_, 1). The inset shows aromatic resonances from the Cy3 fluorophore and the disappearance of the maleimide peak at 7.02 ppm, indicating successful conjugation.

**FIGURE 4:**
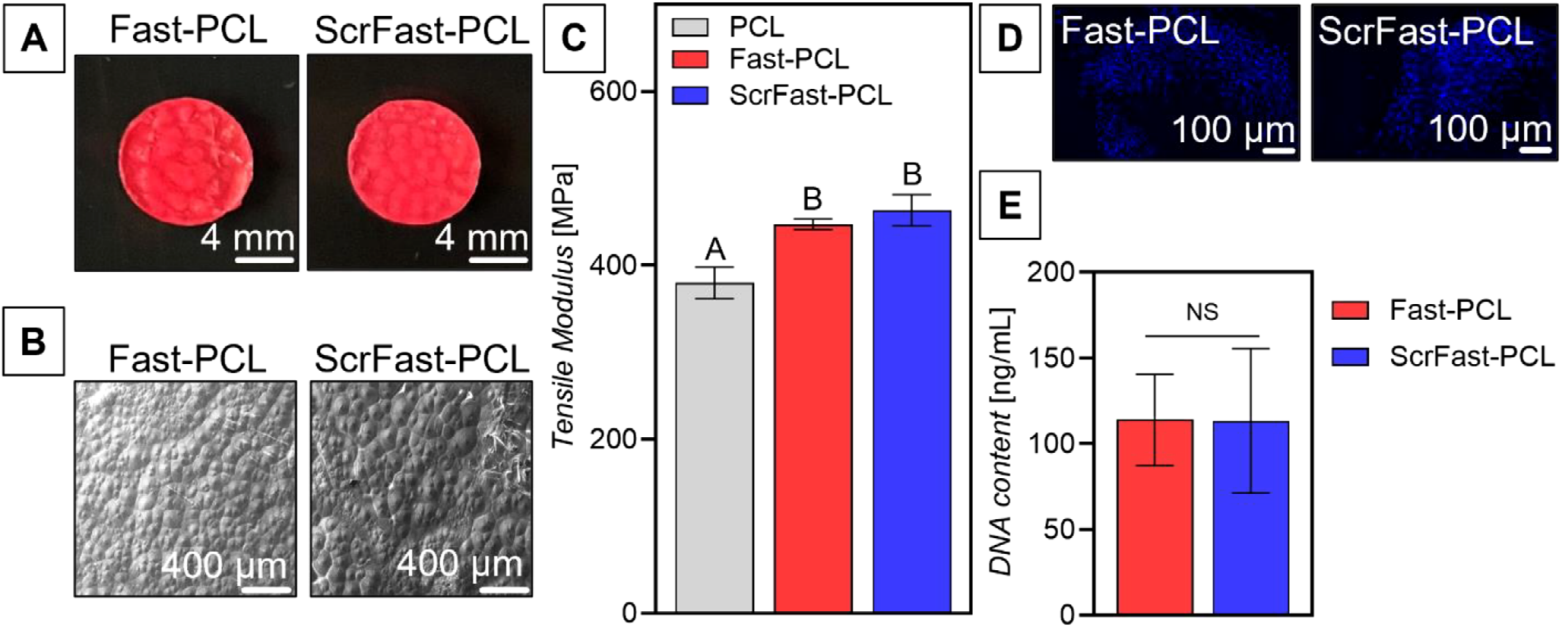
Representative (A) macroscopic and (B) scanning electron microscopy (SEM) images of solvent‐cast samples. (C) Tensile moduli of PCL, Fast‐PCL and ScrFast‐PCL samples. Data presented as mean ± SD (*n* = 3–4 samples per group). P‐values were calculated using one‐way ANOVA followed by Tukey's post‐hoc tests. Different letters indicate statistically significant differences among samples (*p* < 0.05). (D) Representative fluorescence microscopy images of cell nuclei on peptide‐PCL samples stained with Hoechst 33342 (blue). (E) DNA quantification of hMSCs seeded on Fast‐PCL and ScrFast‐PCL samples at Day 4. Data presented as mean ± SD (*n* = 9 samples per group). P‐values were calculated using an independent sample t‐test. NS = not significantly different.

Sample disks were successfully fabricated by solvent‐casting inks containing Fast‐PCL and ScrFast‐PCL conjugates onto glass petri dishes. We included an RGDS‐PCL conjugate in the ink prior to casting to support cell adhesion. We previously showed that RGDS‐PCL can be incorporated prior to scaffold fabrication to functionalize the scaffold surface and promote cell adhesion [71]. RGDS and RGDS‐PCL were successfully synthesized and characterized (Figures S6 and S7) [71]. Both samples appeared bright pink due to the Cy3 fluorophore attached to the peptides (Figure 4A). Representative SEM images revealed similar surface morphologies between Fast‐PCL and ScrFast‐PCL samples, indicating that peptide incorporation did not noticeably alter material topography (Figure 4B). Mechanical testing showed that Fast‐PCL and ScrFast‐PCL exhibited higher tensile moduli than PCL, with no differences between the two modified polymers. Importantly, all tensile modulus values remained within the range reported for bone tissue applications (Figure 4C) [72]. This data also provided complementary evidence supporting successful conjugate synthesis. Both Fast‐PCL and ScrFast‐PCL samples exhibited higher tensile moduli than unmodified 50 kDa PCL, which is consistent with the expected chain length of the PCL‐PEG‐peptide‐PEG‐PCL conjugate. The presence of lower molecular weight species has been shown to reduce mechanical properties due to a decrease in chain entanglements [73, 74, 75, 76]. Fast‐PCL and ScrFast‐PCL samples seeded with hMSCs and stained with Hoechst 33342 showed cell nuclei present on both samples at Day 4 (Figure 4D). DNA quantification indicated no significant differences in the number of cells adhered to each sample (Figure 4E). These results demonstrated that Fast‐PCL and ScrFast‐PCL could be successfully fabricated into similar polymeric biomaterial and that integrating these peptides into the conjugate backbone did not negatively impact cell adhesion.

### 2.2 Sample Degradation with Collagenase

**FIGURE 5:**
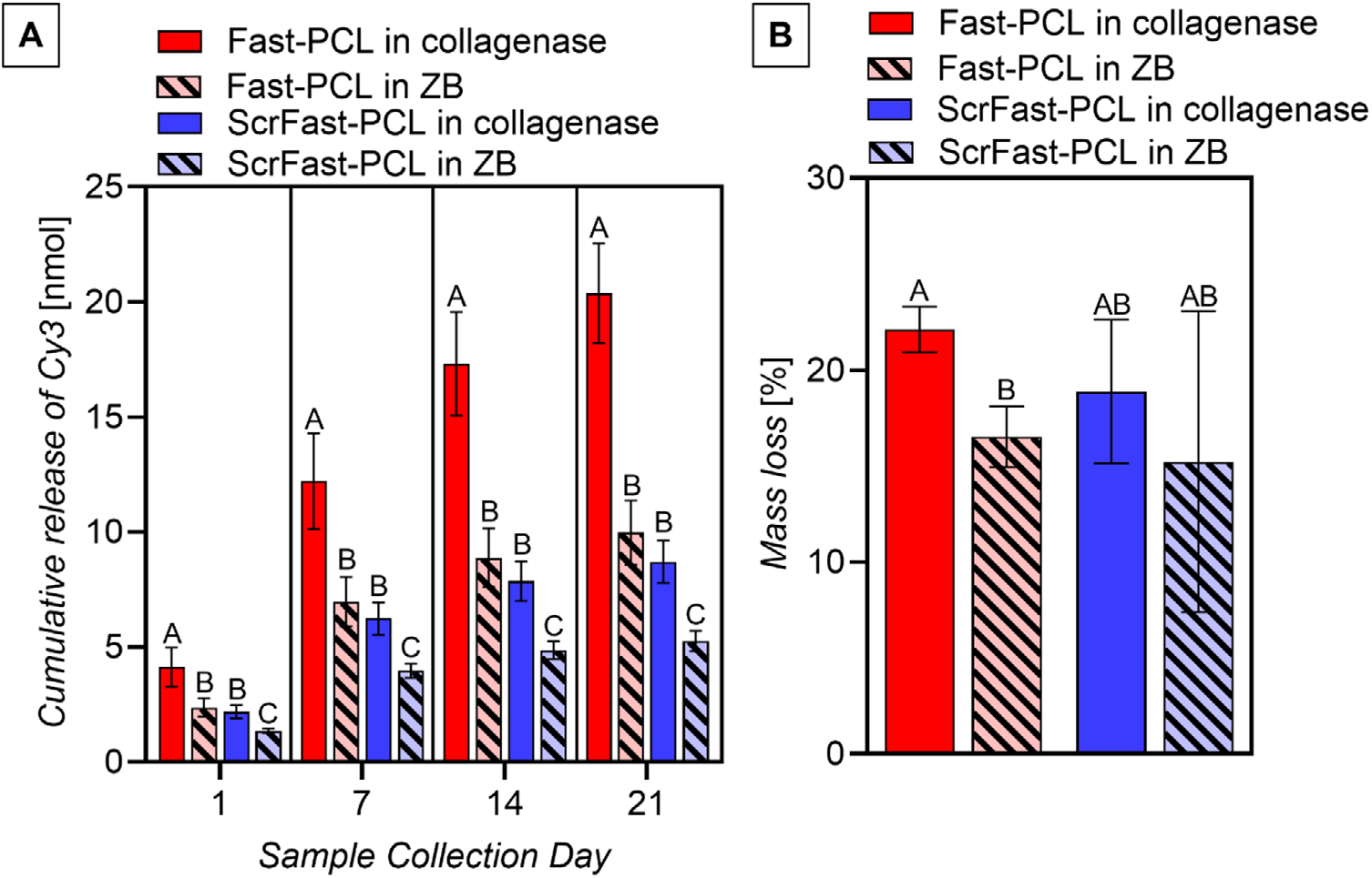
Collagenase‐driven degradation of peptide‐PCL samples. (A) Fluorescence analysis of collected media showed that samples with a fast‐degrading peptide sequence degraded significantly faster than samples with a scrambled sequence of the same amino acids. Data presented as mean ± SD (*n* = 6 samples per group). P‐values were calculated using one‐way ANOVA followed by Tukey's or Dunnett's T3 post‐hoc tests, as appropriate. Comparisons were made across different samples at the same timepoint only. Different letters indicate statistically significant differences among samples within a timepoint (*p* < 0.05). (B) Mass loss of Fast‐PCL and ScrFast‐PCL samples after 21‐day incubation in collagenase or ZB. Data presented as mean ± SD (*n* = 4 samples per group). P‐values were calculated using one‐way ANOVA followed by Dunnett's T3 post‐hoc tests. Different letters indicate statistically significant differences among samples (*p* < 0.05).

The degradation behavior of Fast‐PCL and ScrFast‐PCL samples was assessed under proteolytic conditions to validate the platform design. Sample degradation was quantified via fluorescence intensity of Cy3 released into the media, providing a sensitive and real‐time readout of peptide cleavage within the polymer backbone. We incubated the samples in 2 mg/mL collagenase and monitored degradation for 21 days. Collagenase belongs to the MMP family and is crucial for ECM remodeling, owing to its ability to breakdown collagen, a primary ECM protein [77]. Fluorescence measurements of collected solutions revealed that Fast‐PCL samples degraded significantly faster than ScrFast‐PCL samples in the presence of collagenase (Figure 5A). After 21 days, Fast‐PCL samples showed a cumulative Cy3 release of 20.38 ± 2.17 nmol, equivalent to 25.77% ± 3.70% of the total Cy3 in the sample, while ScrFast‐PCL released only 8.70 ± 0.92 nmol Cy3 release or 11.31% ± 1.01% total Cy3. Both samples in buffer alone exhibited low levels of degradation, likely caused by non‐specific hydrolysis of PCL ester bonds [18]. Interestingly, ScrFast‐PCL samples degraded faster in collagenase compared to buffer alone. The presence of the PEG segments flanking the peptide sequences has been shown to increase hydrophilicity and protease accessibility [62, 63, 64]. The ScrFast peptide showed slight degradation after 24 h in the presence of multiple cell types (Figure 2), indicating some protease susceptibility that would be enhanced by the adjacent PEG spacers. We also observed Fast‐PCL samples in buffer degraded significantly faster than ScrFast‐PCL samples in zymography buffer at all time points. This unexpected result suggests the specific amino acid sequence may impact susceptibility to hydrolytic degradation in the buffer. Together, these results suggested that changes in amino acid sequence impact sample susceptibility to cleavage.

We also measured the masses of Fast‐PCL and ScrFast‐PCL samples before and after 21 days of incubation in collagenase or ZB. Fast‐PCL samples showed a mass loss of 16.5% ± 1.6% in ZB, and 22.1% ± 1.2% in collagenase, representing a significant increase in degradation in the presence of collagenase. In contrast, ScrFast‐PCL samples exhibited mass losses of 15.2% ± 7.8% in ZB, and 18.9% ± 3.8% in collagenase, with no significant difference between conditions (Figure 5B). This mass loss data indicates measurable sample degradation in response to collagenase for Fast‐PCL compared to ScrFast‐PCL samples.

These findings verified that incorporating a protease‐sensitive peptide into the PCL conjugate backbone resulted in protease‐mediated biomaterial degradation. Proteases were able to access and cleave peptides within the peptide‐PCL conjugate backbone. Notably, the data confirmed successful translation of peptide degradation behavior from the peptide screening method to a solid polymeric biomaterial, highlighting sequence‐specific selectivity of protease‐mediated cleavage.

### 2.3 Cell‐Mediated Sample Degradation

Fast‐PCL and ScrFast‐PCL samples were cultured with hMSCs for 10 days. MSCs play a key role in TE because they can be easily isolated from patients for autologous transplantation and are capable of differentiating into nearly all connective tissue phenotypes, including bone, cartilage, skeletal muscles, dense fibrous tissues (i.e. tendons and ligaments), and adipose tissue [78]. In addition, hMSCs express proteases, including MMPs and plasmin, that can proteolytically degrade the peptides that are incorporated into the peptide‐PCL conjugate [79].

**FIGURE 6:**
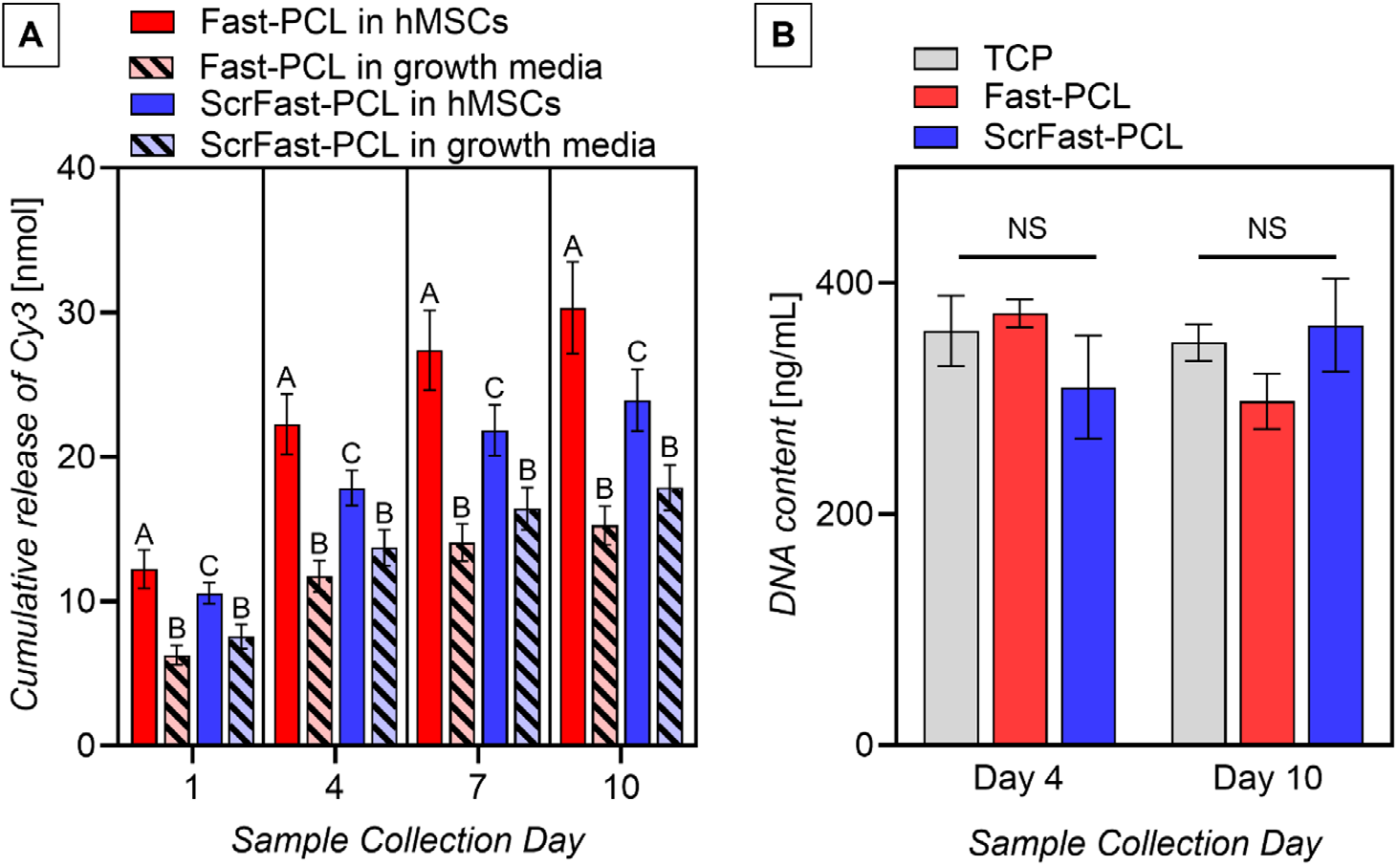
(A) hMSC‐mediated degradation of peptide‐PCL samples. Quantification of Cy3 fluorescence in collected cell culture media showed that samples with the Fast sequence degraded significantly faster than samples with a scrambled sequence (ScrFast) containing the same amino acids. Data presented as mean ± SD (*n* = 5–6 samples per group). P‐values were calculated using one‐way ANOVA followed by Tukey's or Dunnett's T3 post‐hoc tests, as appropriate. Comparisons were made across different samples at the same timepoint only. Different letters indicate statistically significant differences among samples within a timepoint (*p* < 0.05). (B) DNA quantification of hMSCs cultured with Fast‐PCL and ScrFast‐PCL samples at Days 4 and 10 showed no significant differences in cell number between samples and the tissue culture plastic (TCP) control. Data presented as mean ± SD (*n* = 3 samples per group). P‐values were calculated using one‐way ANOVA followed by Tukey's or Dunnett's T3 post‐hoc tests, as appropriate. Statistical analyses were performed separately for each timepoint. NS = not significantly different.

Media samples were collected throughout the culture period to quantify Cy3 release using fluorescence. We observed a higher amount of Cy3 released from Fast‐PCL samples (30.31 ± 3.18 nmol Cy3 release; 26.68% ± 2.17% Cy3 total) compared to ScrFast‐PCL samples (23.91 ± 2.13 nmol Cy3 release; 18.97% ± 1.24% Cy3 total), indicating Fast‐PCL samples degraded significantly faster compared to ScrFast‐PCL samples (Figure 6A). We quantified DNA content for both sample groups compared to a tissue culture plastic (TCP) control at Days 4 and 10 to confirm that the significant differences in sample degradation were not caused by differences in cell number (Figure 6B). In contrast, incubating the samples in growth media without cells showed no measurable differences in degradation between Fast‐PCL and ScrFast‐PCL samples. These data indicated that passive hydrolysis or media components alone did not account for the observed differences (Figure 6A). The Fast peptide was shown to be selectively cleaved by hMSC‐secreted proteases while the scrambled control provided a baseline for nonspecific breakdown.

This study demonstrated that our protease‐cleavable peptide‐PCL conjugates underwent precise, cell‐directed remodeling, establishing a new approach for engineering dynamic, resorbable solid polymeric biomaterials. We presented, to our knowledge, the first quantitative demonstration of cell‐mediated, sequence‐specific degradation in solid polymeric biomaterials by embedding a cleavage motif directly into the polymer backbone. This approach enabled biomaterial degradation to be coupled to protease activity from surrounding cells. The data validated our proteomics‐guided peptide discovery pipeline as a powerful strategy for designing biomaterials with sequence‐specific degradation and position this platform for patient‐tailored regenerative therapies.

## 3 Conclusion

We developed a versatile platform to fabricate solid polymeric biomaterials susceptible to cell‐mediated degradation guided by protease activity. Using a functional mass spectrometry approach, we identified a fast‐degrading peptide sequence (Fast) cleaved by multiple cell types and designed a scrambled control sequence (ScrFast) resistant to proteolysis. Both peptides were integrated into the polycaprolactone (PCL) backbone with PEG linkers to enhance protease accessibility to form linear peptide–PCL conjugates. Incorporating a Cy3 fluorophore into the conjugate enabled real‐time, quantitative tracking of sample breakdown. The Fast‐PCL samples degraded significantly faster than ScrFast‐PCL samples in response to collagenase and hMSCs, confirming that biomaterial remodeling was both sequence‐specific and protease‐driven. These results validated that the peptides remained accessible within the polymer backbone and that biomaterial degradation can be precisely tuned through peptide sequence design, establishing a modular framework for tailoring these biologically responsive materials.

This platform overcomes key limitations with protease‐sensitive hydrogel systems, such as rapid degradation and limited mechanical properties, while retaining the processability and mechanical robustness of thermoplastic materials. The thiol‐Michael addition conjugation strategy used to synthesize the peptide–PCL conjugates can be easily adapted for peptide sequences that target specific tissues and cell types. The conjugates are also compatible with other fabrication techniques, such as 3D printing, to generate patient‐specific, free‐standing 3D‐printed constructs [11, 76, 80, 81]. Such scaffolds are particularly suited for orthopaedic and craniofacial applications where synchronizing implant degradation and [11, 71, 80, 81] bone tissue regeneration is critical for long‐term success. Combining mass spectrometry‐guided peptide discovery with tunable conjugate chemistry establishes a foundation for next‐generation biomaterials with biologically regulated remodeling to advance functional regenerative strategies.

## 4 Experimental Section

### 4.1 Materials

#### 4.1.1 Peptide Synthesis

Materials for peptide synthesis included fluorenylmethyloxycarbonyl chloride (Fmoc)‐protected amino acids from AAPPTec and Fmoc‐L‐Lys(Boc)‐OH sourced from CEM Corporation. Fmoc‐Rink‐amide 4‐methylbenzhydryalmine (MBHA) resin and O─benzotriazole‐N,N,N’,N’‐tetramethyluronium hexafluoro‐phosphate (HBTU) were also obtained from AAPPTec. Piperidine was acquired from BeanTown Chemical while N, N’‐diisopropylethylamine (DIEA), trifluoroacetic acid (TFA), and triisopropylsilane (TIS) came from Sigma‐Aldrich. Other solvents and reagents included diethyl ether (DEE), N,N‐dimethylformamide (DMF), dichloromethane (DCM), and acetonitrile (ACN) from VWR; diisopropylcarbodiimide (DIC) from TCI America; ethyl 2‐cyano‐2(hydroxyamino)acetate (Oxyma) from CEM; and dithiothreitol (DTT) from Gold Biotechnology Inc. The ninhydrin test kit was purchased from Anaspec.

#### 4.1.2 Peptide‐PCL Conjugation

Materials for conjugation included poly(caprolactone) (25 kDa) from Polysciences and amine‐poly(ethylene glycol)‐maleimide (5 kDa) from Advanced BioChemicals (Lawrenceville, GA, USA). Anhydrous N‐methyl pyrrolidone (NMP) was obtained from Alfa Aesar (Ward Hill, MA, USA), while dimethyl sulfoxide (DMSO) was purchased from VWR. Deuterated solvents for NMR analysis included DMSO‐d_6_ and DCM‐d_2_ from Sigma‐Aldrich and DMF‐d_7_ from Thermo Scientific Chemicals.

#### 4.1.3 Biomaterial Fabrication and Sterilization

Biomaterial fabrication utilized 1,1,1,3,3,3‐hexafluoro‐2‐propanol (HFIP) sourced from Matrix Scientific, ethanol from VWR, and phosphate buffer saline (PBS) tablets from Enzo Life Sciences. Additional materials included bovine serum albumin (BSA) from Sigma‐Aldrich and Sylgard 184 silicone encapsulant from Electron Microscopy Sciences.

#### 4.1.4 Degradation with Collagenase

Collagenase Type I (Clostridium histolyticum) from Thermo Scientific Chemicals and Invitrogen Novex Zymogram Developing Buffer (10X) were purchased from Fisher Scientific.

#### 4.1.5 Cell Culture, Staining and DNA Quantification

All cells were obtained commercially from established vendors. THP‐1 monocytes were obtained from ATCC. Human mesenchymal stromal cells (hMSCs) were obtained from RoosterBio, Inc. Fibroblasts from a 27‐year‐old Caucasian female were obtained from Promocell. Cell culture media and supplements included Dulbecco's Modified Eagle's Medium (DMEM; high glucose, GlutaMAX Supplement, pyruvate; Gibco), fetal bovine serum (FBS; GeminiBio), antibiotic‐antimycotic solution containing penicillin, streptomycin, and amphotericin B (anti/anti; Corning), and RPMI Medium (Cytiva).

Other materials included L‐ascorbic acid (Macron Fine Chemicals); interferon‐γ (IFN‐γ), macrophage colony‐stimulating factor (M‐CSF), interleukin‐4 (IL‐4), and interleukin‐13 (IL‐13) from Peprotech; and Gibco TrypLE Express Enzyme (1X), phenol red, and phorbol 12‐myristate 13‐acetate (PMA) from Fisher Scientific. L‐glutamine was purchased from Gibco and lipopolysaccharide (LPS) was from Sigma. For nuclei staining, paraformaldehyde (PFA) was obtained from VWR and Hoechst 33342 from Sigma‐Aldrich. DNA was quantified using an Invitrogen Quant‐iT PicoGreen dsDNA assay kit purchased from Fisher Scientific and tris‐EDTA buffer from Quality Biological.

### 4.2 Methods

#### 4.2.1 Peptide Synthesis and Purification

Peptides (KGPQGIWGQK (PanMMP), KRVKRRLLMET (Fast), PPDKTSPEPA (Control), LTRLMEKRRKV (ScrFast)) were synthesized on Fmoc‐Rink‐amide MBHA resin following standard Fmoc solid‐phase peptide synthesis (SPPS) techniques using a CEM Liberty Blue automated microwave peptide synthesizer. The fast‐degrading and scrambled fast‐degrading peptides were modified with N‐ and C‐terminal cysteine groups and a cyanine3 (Cy3) fluorophore to monitor biomaterial degradation. These peptides were prepared with the following sequences: CK(Cy3)KRVKRRLLMETC (Fast) and CK(Cy3)LTRLMEKRRKVC (ScrFast). All amino acids except for the final two were added using a CEM Liberty Blue automated microwave peptide synthesizer. The cysteine and lysine (protected by a methyltrityl (Mtt) group) at the N‐terminus were coupled manually. The Mtt group was removed using 2% (v/v) TFA and 5% (v/v) TIS in DCM. A water soluble Cy3 was coupled to the epsilon amine in the lysine side chain.

Manual SPPS was performed using a 100 mL peptide synthesis vessel and a wrist action shaker. Fmoc protecting groups were removed with 20% (v/v) piperidine in DMF and the resin was subsequently washed with DMF and DCM. Each amino acid coupling solution was prepared by dissolving 3.95 equivalents of HBTU with 4 molar equivalents of the Fmoc‐protected amino acids in DMF. DIEA was added at six molar equivalents to the amino acid solution before adding to the resin. The reaction was left for at least 3 h then thoroughly washed with DMF and DCM. Ninhydrin tests were conducted to verify successful Fmoc deprotection and amino acid coupling.

Automated SPPS was performed on an automated microwave peptide synthesizer following standard methods developed by CEM. Similar to manual SPPS, a 20% (v/v) piperidine in DMF was used for each deprotection step. Amino acid solutions (4‐fold excess to resin) were prepared by dissolving in DMF. DIC and Oxyma, each at 10 molar equivalents, were used to activate the coupling reactions.

Peptides were cleaved from the resin using 95% (v/v) TFA, 2.5% (v/v) ultrapure water, 2.5% (v/v) TIS, and 2.5% (w/v) DTT. TFA was removed by rotary evaporation. Peptides were recovered by precipitation in cold DEE. The crude peptides were purified using reversed‐phase high performance liquid chromatography (HPLC; Agilent 218 Prep HPLC, Agilent Technologies, Santa Clara, CA, USA). Peptides were dissolved at 10 mg/mL in 95% (v/v) ultrapure water, 4.9% (v/v) ACN and 0.1% (v/v) TFA, sonicated and filtered through a 45 µm PTFE syringe filter to remove particulates. Samples were purified through an Agilent 5 Prep‐C18 column (150 x 21.2 mm, 5 µm pore size, 100 Å particle size) using a mobile phase composed of ACN with 0.1% TFA and ultrapure water with 0.1% TFA. Fractions containing the desired peptide were pooled and subjected to rotary evaporation to remove ACN and TFA before lyophilization. The mass of purified peptides was confirmed by matrix‐assisted laser desorption/ionization time of flight mass spectrometry (MALDI TOF‐MS; Shimadzu Benchtop MALDI‐ToF 8020).

#### 4.2.2 Peptide Degradation Studies with Multiple Cell Types

We identified a peptide KRVKRRLLMET (Fast) that exhibited rapid proteolytic cleavage after 24 h with multiple cell types based on prior peptide screening methods [66]. We compared its degradation behavior to a peptide that showed negligible degradation behavior (PPDKTSPEPA; Control) and a well‐established MMP‐degradable peptide (GPQGIWGQ; PanMMP). We also included a scrambled version of Fast (LTRLMEKRRKV; ScrFast) in which the positions of uncharged amino acids (L, M, V) were exchanged with the charged amino acids (E, K, R). We quantified degradation of these four peptides with different macrophage phenotypes, hMSCs, and fibroblasts.

To generate macrophages, THP‐1 cells were placed into a 48 well plate with a seeding density of 500 000 cells per well using 500 µL of RPMI containing 10% FBS and 1% anti‐anti, with PMA at a concentration of 100 ng/mL for two days to generate M0 macrophages. To generate M1 macrophages, the media was changed after Day 2 to RPMI containing interferon‐γ (IFN‐γ), 20 ng/mL macrophage colony‐stimulating factor (M‐CSF), and 100 ng/mL lipopolysaccharide (LPS) and allowed to polarize for three days (Day 5 of culture). To generate M2A macrophages, the media was changed after Day 2 to RPMI containing 20 ng/mL M‐CSF, 40 ng/mL of interleukin‐4 (IL‐4), and 20 ng/mL of interluekin‐13 (IL‐13). To generate M2C macrophages, the media was changed after Day 2 to RPMI containing 20 ng/mL M‐CSF and 20 ng/mL of interluekin‐10 (IL‐10). For degradation studies the media was changed to macrophage serum free media with L‐glutamine. Macrophage polarization was validated using established marker expression analysis. Detailed methods and results (Figure S8) are provided in the Supplementary Information.

hMSCs (36 000 per well), fibroblasts (30 000 per well) and THP‐1 derived macrophages (500 000 cells per well) were seeded in 48‐well plates with 500 µL media added per well and cultured for 24 h. Each of the peptides was added to the media to generate a 50 µM concentration and a non‐proteolytically degradable NH_2_‐βFβAβAβAβAβAβA‐amide peptide (βFβA_6_), where βF is β‐phenylalanine and βA is β‐alanine, was added at a 50 µM concentration as used as an internal standard. Conditioned media (100 µL) was sampled at 0 and 24 h. Acetic acid (4 µL) was added to all collected media in the 96 wells LC‐MS plates and they were stored in a −80 °C freezer until analysis to prevent further proteolytic degradation. The samples were then thawed and analyzed by liquid chromatography‐mass spectrometry (LC‐MS). Peptides degradation ratios were measured by LC‐MS (Thermo Fisher Vanquish UPLC, LTQ‐XL mass spectrometer) and analyzed by Xcalibur. The relative concentrations of peptide were calculated by the ratio of peak area of peptide and peak area of βFβA_6_.

#### 4.2.3 Synthesis of Peptide‐PCL Conjugates

Polycaprolactone (25 kDa; HO‐PCL‐COOH) was modified with amine‐poly(ethylene glycol)‐maleimide (5 kDa; amine‐PEG‐mal) following an amide coupling process. PCL (125 mg/mL) was dissolved in anhydrous NMP. HBTU was added as an activator at a mol ratio of 4:3.95 PCL:HBTU. DIEA was added to the PCL/activator solution at a molar ratio of 4:6 PCL:DIEA to activate the carboxyl end of PCL. Amine‐PEG‐mal dissolved in 50:50 NMP:DMSO at 2 molar equivalents to PCL was added dropwise to the activated PCL solution while stirring at 350 rpm. The resulting solution was purged with N_2_ for 15 min and left to stir overnight while protected from light. Maleimide‐terminated PCL (PCL‐PEG‐mal) was recovered by trituration with room temperature DEE.

Peptide‐PCL (Fast‐PCL and ScrFast‐PCL) conjugates were synthesized via maleimide‐thiol conjugation between PCL‐PEG‐mal and the thiol‐terminated peptides. PCL‐PEG‐mal was dissolved in anhydrous NMP at 2 molar equivalents to the peptide. The peptide was separately dissolved in anhydrous NMP then added dropwise to the PCL‐PEG‐mal solution while stirring at 500 rpm. The resulting solution was purged with N_2_ for 15 min and continued to react overnight while protected from light. Fast‐PCL and ScrFast‐PCL conjugates were obtained by trituration with cold DEE. RGDS‐PCL conjugate was also prepared using established methods previously described [68]. Each synthesis was confirmed by proton nuclear magnetic resonance (^1^H NMR) and diffusion‐ordered spectroscopy (DOSY). NMR samples were prepared by dissolving in either DCM‐d_2_, DMSO‐d_6_ or DMF‐d_7_ and analyzed using a 500 mHz NMR spectrometer (Bruker). Diffusion measurements were acquired with ledbpgp2s pulse program. Data processing was performed using MestReNova software (v14.3.3‐33362).

#### 4.2.4 Biomaterial Fabrication

Inks were prepared by mixing Fast‐PCL or ScrFast‐PCL (195 mg/mL) and RGDS‐PCL (5 mg/mL) conjugates in HFIP. Inks were shaken on a wrist‐action shaker at room temperature for 48 h then allowed to rest at room temperature for 24 h. Inks were cast onto the top lid of a 60 mm glass petri dish and allowed to dry in the hood for approximately 45 min. To ensure flatness, the bottom lid was placed over the dried ink, taped, and left to dry overnight. Samples were then punched into 10 mm disks using a biopsy punch. The disks were immersed in 75% ethanol for 30 min, rinsed three times with sterile ultrapure water, soaked in 0.1% BSA for at least four hours, and subsequently rinsed three times with sterile PBS. The samples were left to dry inside a biosafety hood before use.

#### 4.2.5 SEM Characterization

Sample surfaces were characterized using a scanning electron microscope (SEM; Axia ChemiSEM, ThermoFisher Scientific). Samples were mounted on 12‐mm aluminum stubs using carbon tape then coated with iridium using a sputter coater (Electron Microscopy Sciences). Sample images were obtained using a secondary electron detector with an accelerating voltage of 5 kV.

#### 4.2.6 Tensile Test

Strips (40 mm x 1 mm) were cut from solvent‐casted solutions of 195 mg/mL 50 kDa PCL, Fast‐PCL, or ScrFast‐PCL conjugates and 5 mg/mL RGDS‐PCL. Tensile properties were measured using a KLA Universal Testing Machine T150. Each strip was mounted on a cardstock frame with a 20 mm gauge length and extended at a strain rate of 0.001 s^−1^ until a maximum strain of 0.025 mm/mm was reached.

#### 4.2.7 Cell Adhesion Assay

Human MSCs from a 25‐year‐old Caucasian male donor were used to assess cell adhesion on Fast‐PCL and ScrFast‐PCL samples. These cells were cultured in DMEM‐GlutaMAX supplemented with 10% (v/v) FBS, 1% (v/v) anti/anti solution, and 0.1% L‐ascorbic acid and maintained at 37°C in 5% CO_2_. Cells were expanded to 80%–90% confluency and subsequently harvested using Gibco TrypLE Express Enzyme (1X), phenol red. Passages 3–4 were selected for cell seeding. Cells (500,000 cells/mL; 100 µL) were seeded on top of the pinned samples and incubated at 37°C and 5% CO_2_ to allow for cell adhesion. After one hour, growth media was added in each well to reach a final volume of 1 mL. Samples were harvested and analyzed for fluorescence imaging on Day 4. Culture media was removed from the wells and samples were rinsed twice with sterile PBS. Samples were fixed in 4% (w/v) PFA in PBS for 1 h at 4°C then washed two times in PBS. All samples were stored in PBS at 4°C until stained. Prior to staining, cells were permeabilized using 0.1% (v/v) Triton X‐100 in PBS for 15 min. Hoechst 33342 (10 mg/mL in ultrapure water) diluted 1:5000 in ultrapure water was added for nuclear staining. Samples were washed twice with PBS and stored in PBS at 4°C protected from light until imaging. Fluorescence images were acquired using a Keyence BZ‐X810 microscope.

#### 4.2.8 In Vitro Degradation with Collagenase

Samples were pinned to silicone elastomer‐coated 24‐well plates using 0.1 mm dissection pins. The degradation behavior of the samples in the presence of collagenase was assessed by immersing pinned samples in 2 mg/mL collagenase in 1X Zymogram Developing Buffer (ZB). Solutions (1 mL) were collected and changed after the first day of incubation then after every 3–4 days. Samples were placed in an incubator (37°C, 5% CO_2_) for up to 21 days. Samples were also exposed to ZB as a baseline control.

#### 4.2.9 Mass Loss Measurements

Samples were weighed before and after 21 days of incubation with ZB or collagenase. After the 21‐day incubation period, samples were collected and washed three times with ultrapure water to remove residual enzymes or salts. Following washing, samples were lyophilized and subsequently weighed to determine the final mass. Percent mass loss was calculated based on the difference between initial and final masses.

#### 4.2.10 In Vitro Degradation with hMSCs

Human MSCs were cultured as described above to assess cell‐mediated degradation of Fast‐PCL and ScrFast‐PCL samples in vitro. Cells were seeded at passage 3–4 into 24‐well plates at 50 000 cells/well in 100 µL and incubated at 37°C with 5% CO_2_ for one hour to allow for cell adhesion. Media was added to each well to a final volume of 1 mL per well. Culture media was replaced after the first day and then every three days to monitor degradation for up to 10 days. Samples were also immersed in growth media alone as a baseline control.

#### 4.2.11 DNA Quantification

DNA content was analyzed on Days 4 and 10. After collecting media, samples were carefully rinsed twice with sterile PBS. Subsequently, 0.2% Triton X‐100 (0.5 mL) was added to each well, and cells were scraped and thoroughly mixed using a pipette. The resulting solutions were transferred to Eppendorf tubes and stored at −80°C until further analysis. DNA quantification was carried out using the PicoGreen assay, with fluorescence measurements taken at excitation and emission wavelengths of 485 and 520 nm, respectively, using an Infinite M Nano Tecan microplate reader. DNA ladder (0 −1000 ng/mL) was prepared using calf thymus DNA in 1X Tris‐EDTA buffer.

#### 4.2.12 Fluorescence Analysis

The degradation of Fast‐PCL and ScrFast‐PCL samples was assessed by quantifying the Cy3 released into the solution. Fluorescence intensity was measured with excitation and emission wavelengths set at 550 and 580 nm, respectively, using an Infinite M Nano Tecan microplate reader (Tecan Austria GmbH). A standard curve (0–2 nmol Cy3) was created using Cy3 dissolved in ZB, collagenase in ZB, or hMSC growth media to calculate the Cy3 released by the samples in different conditions.

#### 4.2.13 Statistical Analysis

All quantitative data are presented as mean ± SD. Independent samples t‐tests were used to assess cell attachment on Fast‐PCL and ScrFast‐PCL samples (*n* = 9 samples per group). Statistical significance for this experiment was defined as ^*^ *p* < 0.05, ^**^p < 0.01, and ^***^ *p* < 0.001. For comparisons involving more than two groups, one‐way analysis of variance (ANOVA) was applied, followed by Tukey's or Dunnett's T3 post‐hoc tests, as appropriate. This analysis was used to compare degradation behavior of peptides in different cell types (*n* = 3–8 samples per group), tensile properties (*n* = 3–4 samples per group), mass loss (*n* = 4 samples per group), relative fluorescence intensity of solutions collected at different time points (*n* = 5–6 samples per group) and DNA content per timepoint (*n* = 3 samples per group). Groups not sharing the same letter were considered significantly different (*p* < 0.05). Statistical analysis was performed using SPSS (IBM, version 31.0.0.0).

## Author Contributions

K.V.G.S. did the study design, performed experiments, data analysis and interpretation, wrote the original draft of the manuscript, and reviewed and edited the final manuscript. N.K.H. conceptualized the project, performed preliminary experiments, and contributed to manuscript editing. S.S. conceptualized the project, contributed to manuscript editing, and provided critical review comments. K.B.S. conceptualized the project and performed preliminary experiments. Y.W. performed experiments, data analysis and interpretation, and contributed to manuscript writing and editing. E.T.P. conceptualized the project, performed experiments, data analysis, and interpretation, contributed to manuscript writing, and provided critical review comments. W.L.G. conceptualized the project, helped with data analysis and interpretation, contributed to manuscript editing, and provided critical review comments. L.W.C. conceptualized and supervised the project, secured funding, performed data analysis and interpretation, contributed to manuscript writing, and provided critical review comments. All authors reviewed and approved the final version of the manuscript.

## Conflicts of Interest

The authors declare no conflicts of interest.

## Supporting information




**Supporting File**: adhm70970‐sup‐0001‐SuppMat.docx

## Data Availability

The data that support the findings of this study are available from the corresponding author upon reasonable request.
