# Supplementary material for: Design and Synthesis of Peptide‐Polyester Conjugates for Cell‐Mediated Scaffold Degradation
Source: Adv Healthc Mater. 2026 Feb 21;15(27):e04885. doi: 10.1002/adhm.202504885 (PMC13378486; doi:10.1002/adhm.202504885)
Supplement: Supplementary file 1 — Supporting File: adhm70970‐sup‐0001‐SuppMat.docx [file ADHM-15-0-s001.docx]

**Supplementary Information**

**Title:** Design and Synthesis of Peptide-Polyester Conjugates for Cell-Mediated Scaffold Degradation

**Authors:** *Korina Vida G. Sinad^1^, Natasha K. Hunt^2^, Srujan Singh^3,4^, Kelly B. Seims^5^, Yingjie Wu^2^, E. Thomas Pashuck^2^, Warren L. Grayson^3,4,6,7,8^, Lesley W. Chow^2,5,9^**

*Corresponding Author

Email: [lac415@lehigh.edu](mailto:lac415@lehigh.edu)

^1^Department of Chemistry, Lehigh University, Bethlehem, PA, USA

^2^Department of Bioengineering, Lehigh University, Bethlehem, PA, USA

^3^Department of Chemical and Biomolecular Engineering, Johns Hopkins University, Baltimore, MD, USA

^4^Translational Therapeutics & Regenerative Engineering Center, Johns Hopkins University School of Medicine, Baltimore, MD, USA

^5^Department of Materials Science & Engineering, Lehigh University, Bethlehem, PA, USA

^6^Department of Biomedical Engineering, Johns Hopkins University, Baltimore, MD, USA

^7^Institute for Nanobiotechnology, Johns Hopkins University, Baltimore, Maryland, USA

^8^Department of Materials Science and Engineering, Johns Hopkins University, Baltimore, MD, USA
^9^College of Health, Lehigh University, Bethlehem, PA, USA


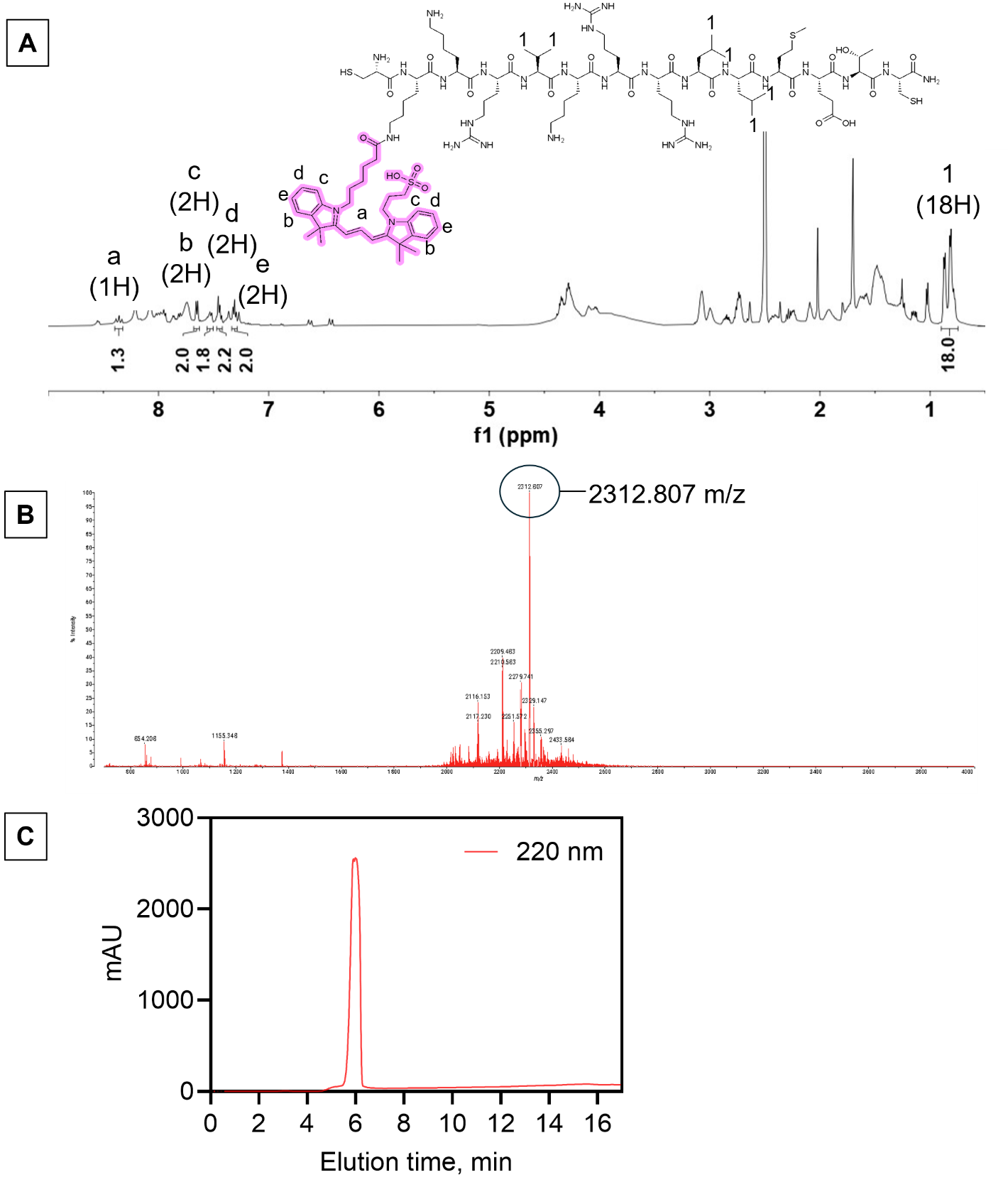


**Figure S1.** Purified CK(Cy3)KRVKRRLLMETC. Representative (A) ^1^H NMR spectrum (500 MHz, DMSO-d_6_) and corresponding chemical structures: δ = 8.36 (t, 1H, vinyl H, a), 7.65 (d, 2H, Ar-H, b), 7.53 (d, 2H, Ar-H, c), 7.45 (m, 2H, Ar-H, d), 7.31 (t, 2H, Ar-H, e), and 0.81 (m,18H, -CH_3_, 1), and (B) MALDI-ToF MS spectrum (MW 2311 Da), and (C) HPLC profile.


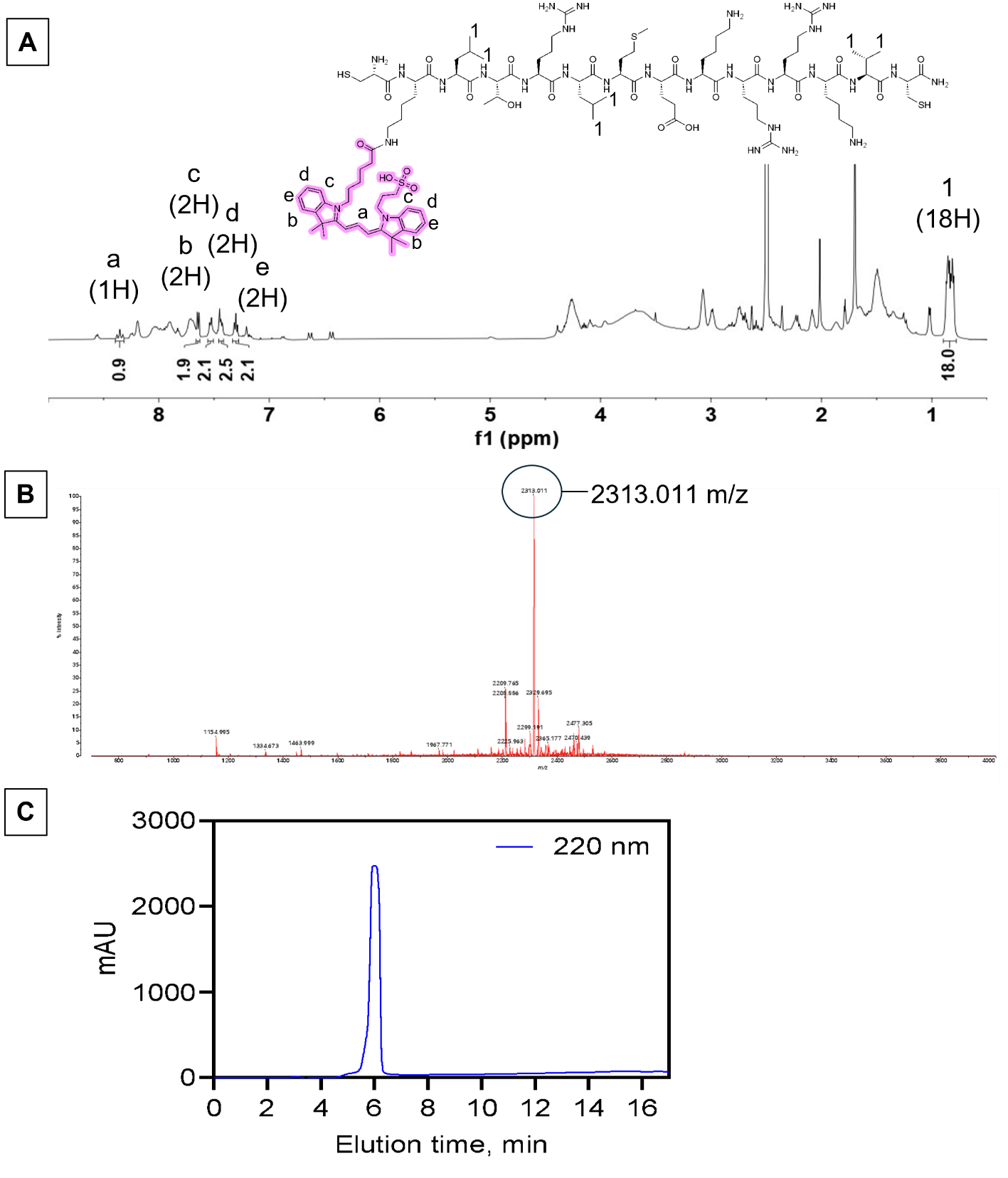


**Figure S2.** Purified CK(Cy3)LTRLMEKRRKVC. Representative (A) ^1^H NMR spectrum (500 MHz, DMSO-d_6_) and corresponding chemical structures: δ = 8.35 (t, 1H, vinyl H, a), 7.64 (d, 2H, Ar-H, b), 7.53 (d, 2H, Ar-H, c), 7.44 (m, 2H, Ar-H, d), 7.30 (t, 2H, Ar-H, e), and 0.84 (m,18H, -CH_3_, 1), and (B) MALDI-ToF MS spectrum (MW 2311 Da), and (C) HPLC profile.


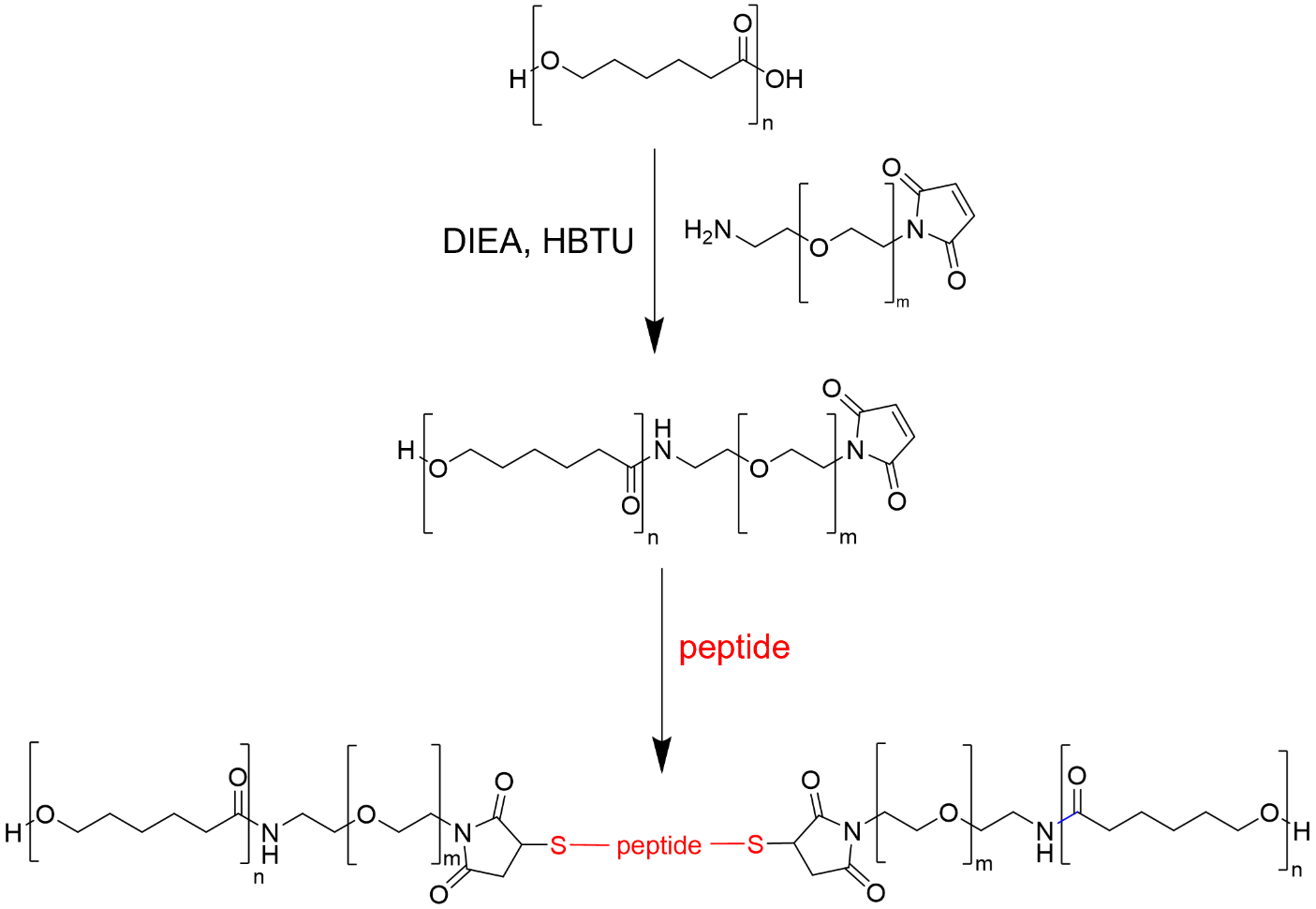


**Figure S3.** Peptide-PCL conjugation reaction scheme. Peptide was either a modified fast-degrading peptide sequence (Fast; CK(Cy3)KRVKRRL↓LMETC) or a scrambled version (ScrFast; CK(Cy3)LTRLMEKRRKVC).

**
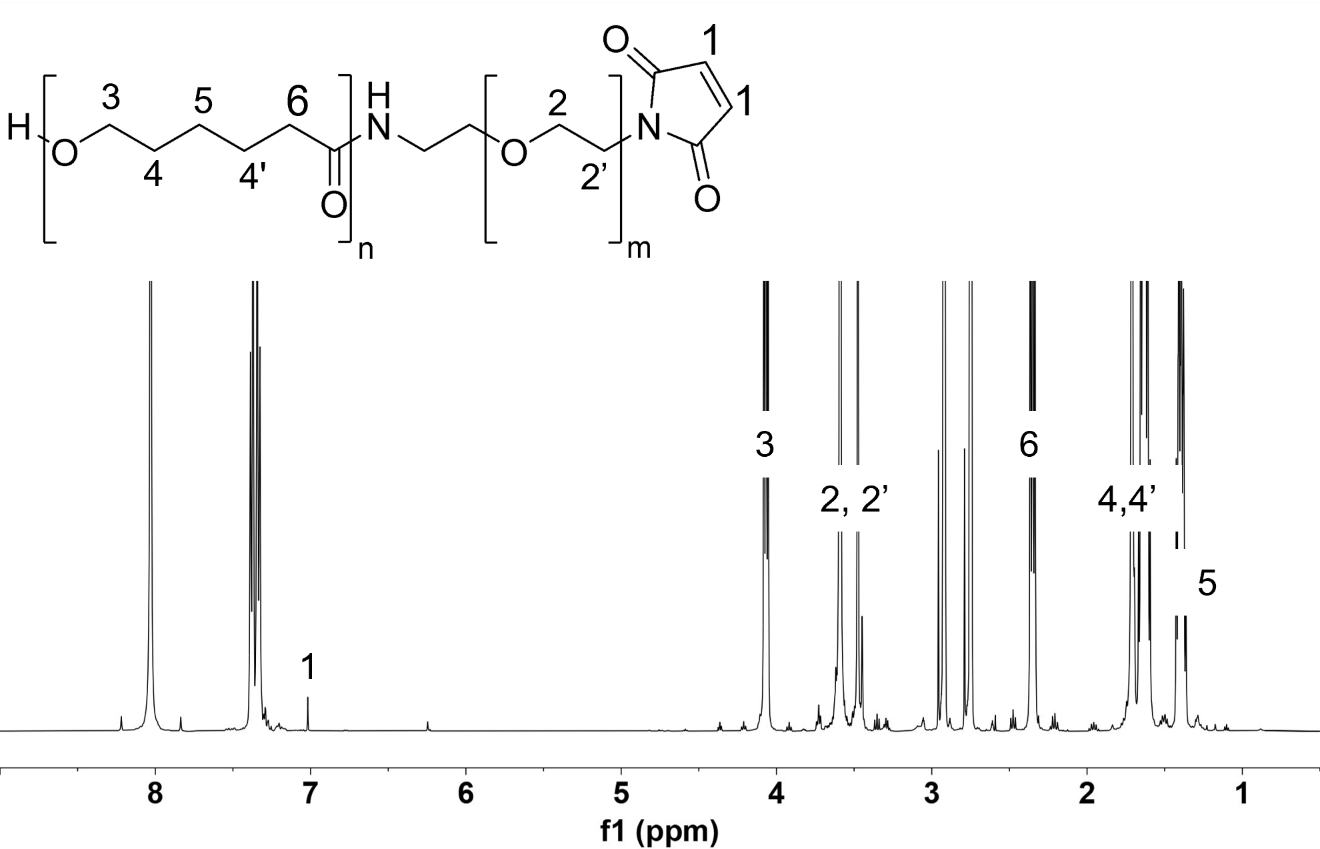
**

**Figure S4.** Representative ^1^H NMR spectrum (500 MHz, DMF-d_7_) and corresponding chemical structures of maleimide-functionalized PCL: δ = 7.02 (s, 2H, maleimide vinyl H, 1), 3.59 (s, 436H, -O-CH_2_-CH_2_-, 2/2’), and 4.07 (t, 456H, -O-CH_2_-, 3), 2.35 (t, 456H, -CO-CH_2_-, 6), 1.62 (m, 912H, -CH_2_-, 4/4’), and 1.39 (m, 456H, -CH_2_-, 5).

**
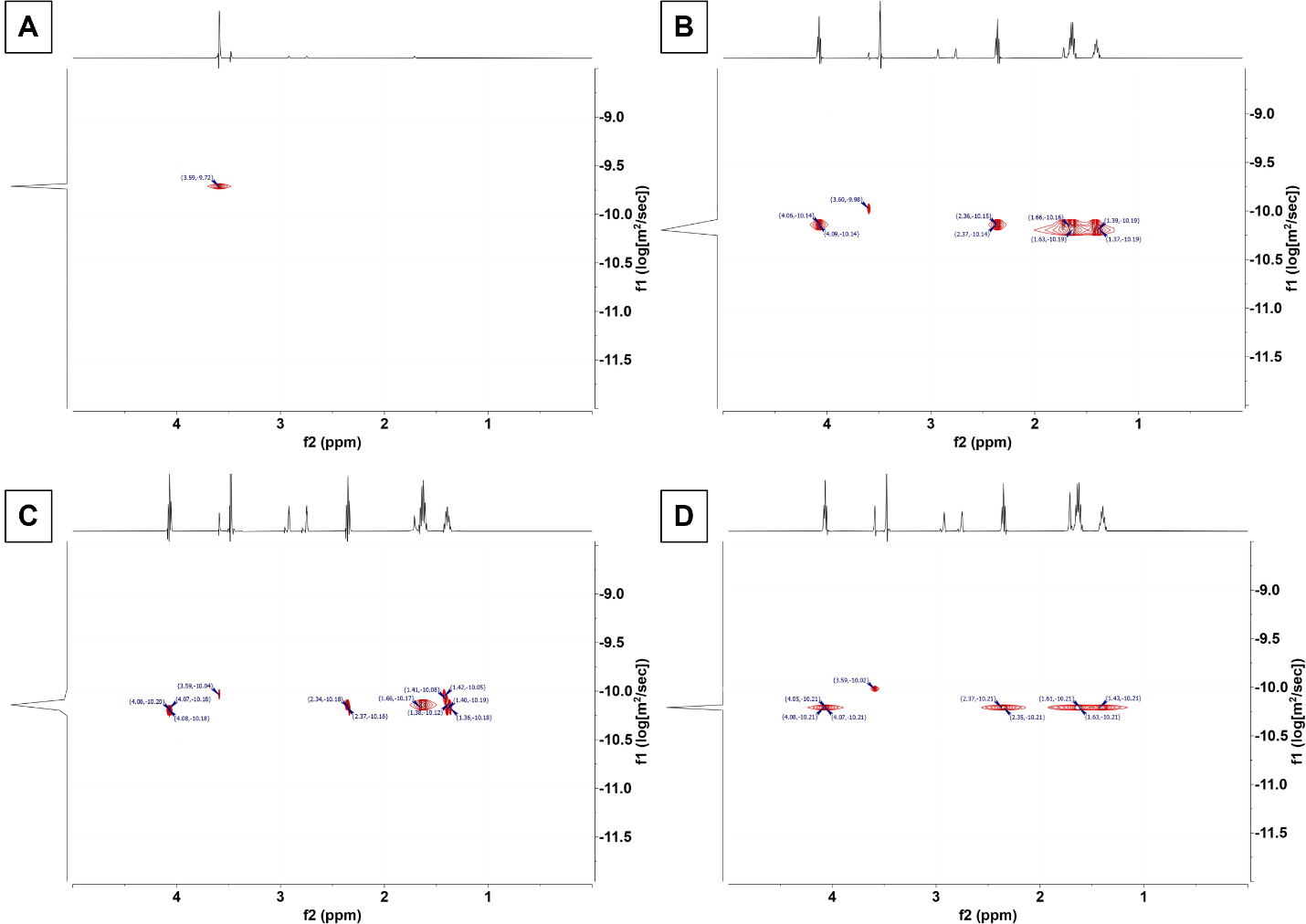
**

**Figure S5.** Representative ^1^H DOSY NMR spectra (500 MHz, DMF-d_7_): (A) amine-PEG-maleimide, (B) PCL-PEG-mal, (C) Fast-PCL, and (D) ScrFast-PCL conjugate.


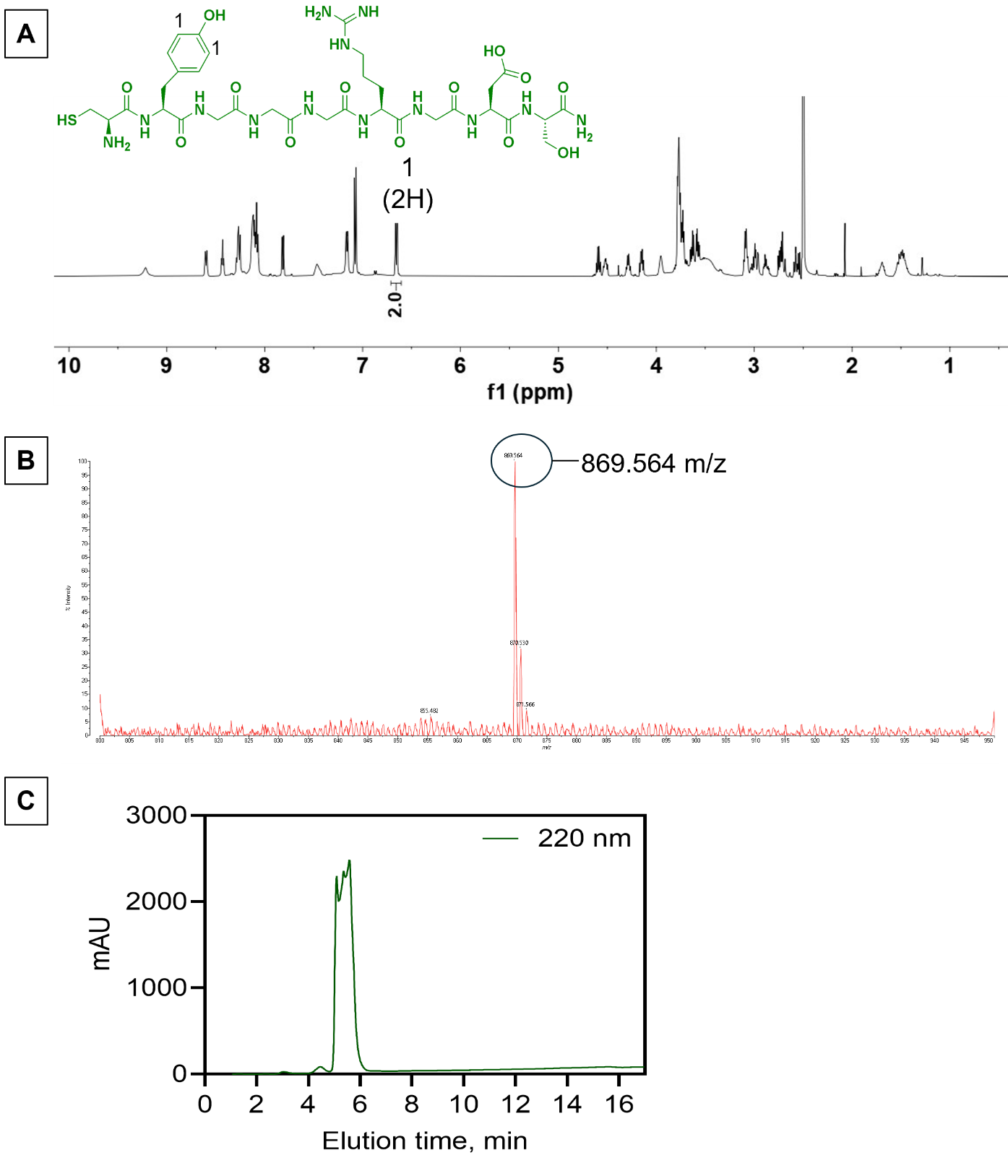


**Figure S6.** Purified CYGGGRGDS (RGDS). Representative (A) ^1^H NMR spectrum (500 MHz, DMSO-d_6_) and corresponding chemical structures with chemical shift assignments: 6.65 (d, 2H, Ar-H, 1), (B) MALDI-ToF MS spectrum (MW 869 Da), and (C) HPLC profile.


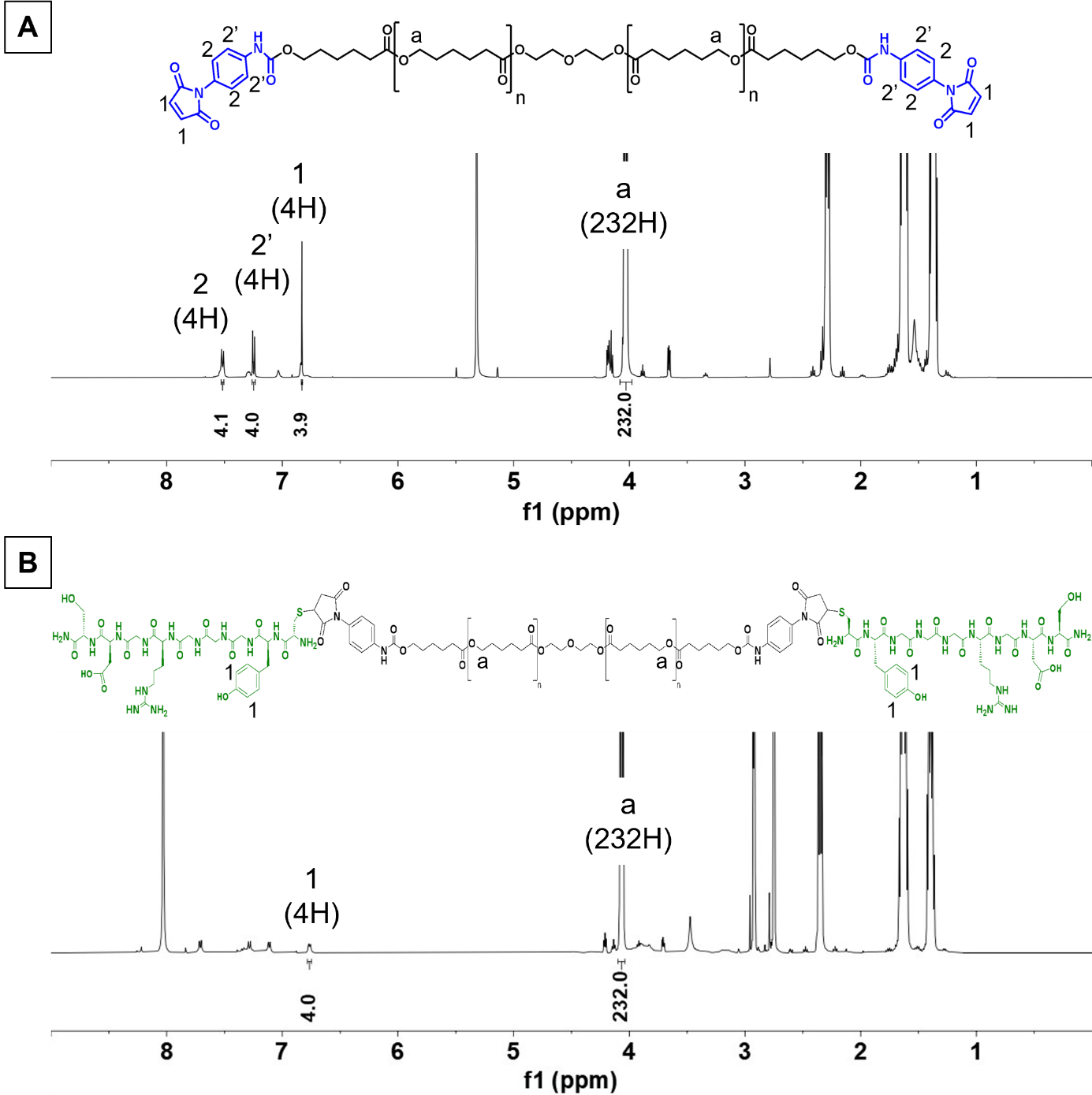


**Figure S7.** Representative ^1^H NMR spectrum (500 MHz) and corresponding chemical structures with chemical shift assignments of (A) PCL-maleimide (DCM-d_2_): δ = 6.83 (s, 4H, maleimide vinyl, 1), 7.25 (d, 4H, Ar-H ortho to isocyanate, 2’), 7.52 (d, 4H, Ar-H ortho to maleimide, 2), and 4.03 (t, 232H, -O-CH_2_-, a) and (B) RGDS-PCL (DMF-d_7_): δ = 6.77 (d, 4H, Ar-H, 1) and 4.07 (t, 232H, -O-CH_2_-, a).

**Quantitative real-time PCR.** To validate macrophage phenotype differentiation, quantitative real-time PCR was performed. THP-1–derived macrophage polarization was performed according to the method described above. Total RNA was isolated from 4 types of macrophages (M0, M1, M2A and M2C) using the Quick-RNA™ Plant MiniPrep Kit (Zymo Research Corporation). RNA concentration and purity were assessed using a SpectraMax iD3 Multi-Mode Microplate Reader (Molecular devices). Complementary DNA (cDNA) was synthesized from isolated RNA using the ImProm-II™ Reverse Transcription System (Promega). Quantitative real-time PCR (qPCR) was performed using PerfeCTa® SYBR® Green SuperMix, Low ROX (Quantabio) with gene-specific forward and reverse primers for IL1b, MRC1, TNF, CCL18, SOCS3, CXCL-10, TGFB, and CD163 (Integrated DNA Technologies). Amplification and detection were carried out on a QuantStudio™ 3 Real-Time PCR System (Applied Biosystems). Relative mRNA expression levels were calculated using the 2^−ΔΔCt^ method, with RPS18 used as the endogenous housekeeping control gene. All procedures for RNA isolation, cDNA synthesis, and qPCR analysis were conducted following the manufacturer’s instructions.

**
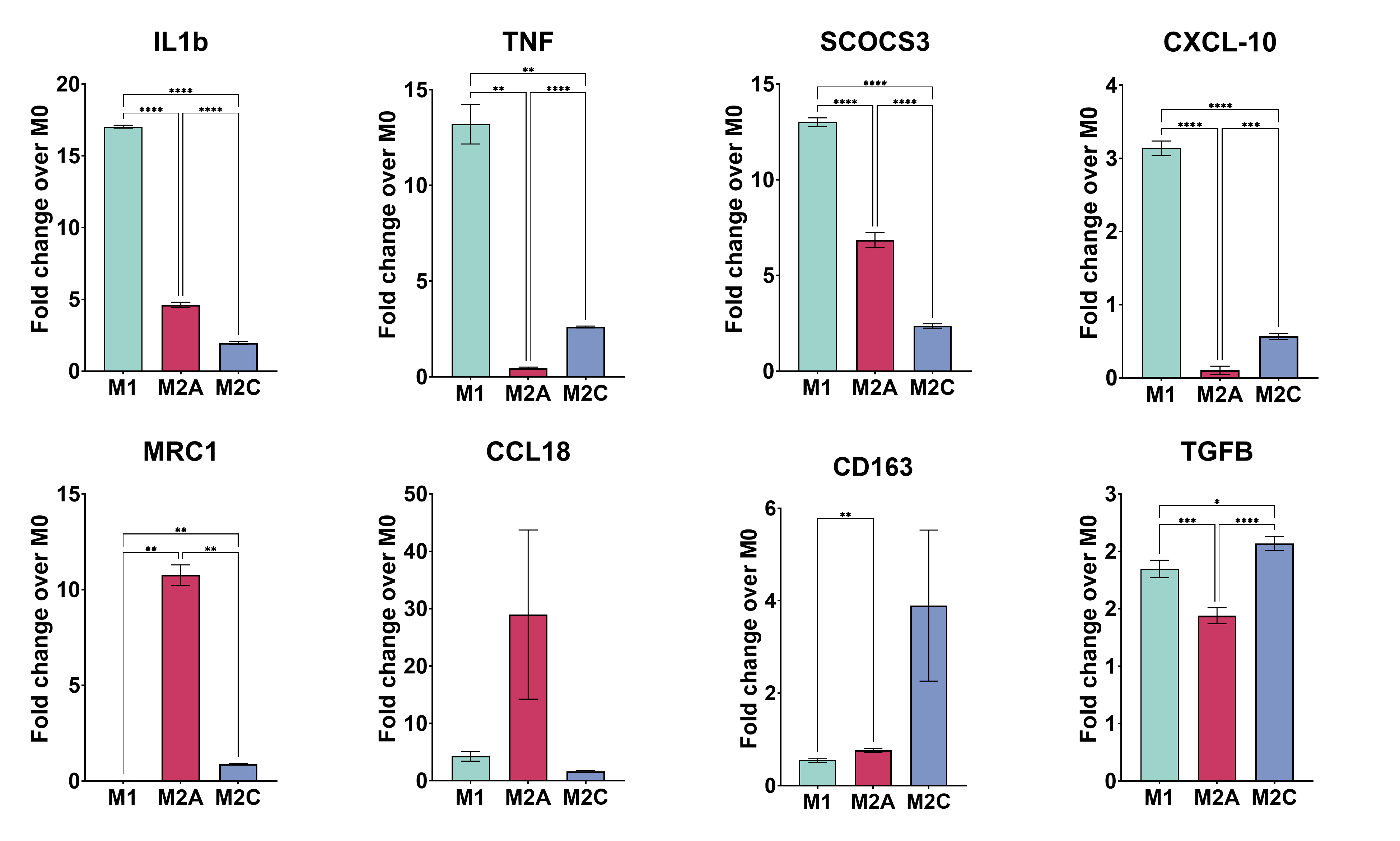
**

**Figure S8.** Gene expression levels of M1- and M2-associated markers validate macrophage phenotype. Data presented as mean ± SD (n = 3 samples per group). P-values were calculated using one-way ANOVA followed by Tukey’s or Dunnett’s T3 post-hoc tests, as appropriate. Significance levels: *p < 0.05, **p < 0.01, ***p < 0.001.
